# The Macaque Social Responsiveness Scale (mSRS): A Rapid Screening Tool for Assessing Variability in the Social Responsiveness of Rhesus Monkeys (*Macaca mulatta*)

**DOI:** 10.1371/journal.pone.0145956

**Published:** 2016-01-05

**Authors:** Eric J. Feczko, Eliza Bliss-Moreau, Hasse Walum, John R. Pruett, Lisa A. Parr

**Affiliations:** 1 Yerkes National Primate Research Center, Atlanta, GA 30329, United States of America; 2 Center for Translational Social Neuroscience, Emory University, Atlanta, GA 30329, United States of America; 3 Department of Psychiatry and Behavioral Science, California National Primate Research Center, University of California, Davis CA 95616, United States of America; 4 Department of Psychiatry, Washington University, School of Medicine, St. Louis, MO 63110, United States of America; 5 Department of Psychiatry and Behavioral Science, Emory University, Atlanta, GA 30322, United States of America; Institut Pluridisciplinaire Hubert Curien, FRANCE

## Abstract

Understanding the biological mechanisms underlying human neuropsychiatric disorders, such as autism spectrum disorder (ASD), has been hindered by the lack of a robust, translational animal model. Rhesus monkeys (*Macaca mulatta*) display many of the same social behaviors that are affected in ASD, making them an excellent animal species in which to model social impairments. However, the social impairments associated with ASD may reflect extreme ends of a continuous distribution of traits. Thus, to validate the rhesus monkey as an animal model for studying social impairments that has strong translational relevance for ASD, researchers need an easily-implemented measurement tool that can quantify variation in social behavior dimensionally. The Social Responsiveness Scale (SRS) is a 65-item survey that identifies both typical and atypical social behaviors in humans that covary with ASD symptom severity. A chimpanzee SRS has already been validated and the current study adapted this tool for use in the rhesus monkey (mSRS). Fifteen raters completed the mSRS for 105 rhesus monkeys living at the Yerkes National Primate Research Center. The mSRS scores showed a unimodal distribution with a positive skew that identified 6 statistical outliers. Inter-rater reliability was very strong, but only 17 of the 36 questions showed positive intra-item reliability. The results of an exploratory factor analysis identified 3 factors that explained over 60% of the variance, with 12 items significantly loading onto the primary factor. These items reflected behaviors associated with social avoidance, social anxiety or inflexibility and social confidence. These initial findings are encouraging and suggest that variability in the social responsiveness of rhesus monkeys can be quantified using the mSRS: a tool that has strong translational relevance for human disorders. With further modification, the mSRS may provide an promising new direction for research on the biological mechanisms underlying social impairments.

## Introduction

Autism Spectrum Disorder (ASD) is highly prevalent in the human population, estimated to occur in 1 in 68 individuals in the United States (www.cdc.gov). Historically, ASD has been characterized by three core features; language impairments, social interaction deficits, and an increased occurrence of repetitive or restricted behaviors and interests. Despite urgent need, there are currently no approved FDA treatments for the core symptoms of ASD. While understanding the basic biological mechanisms underlying ASD is critical for the development of effective treatments, advances have been limited, in part, because the clinical symptomatology of ASD is highly variable, and there is broad genetic heterogeneity across individuals [1–2]. Moreover, basic science focused on understanding the biological mechanisms underlying the social impairments associated with ASD and related disorders has been severely hindered by the lack of a robust, translational animal model. Developing an animal model of social impairments could significantly advance our understanding of ASD and other neuropsychiatric conditions. In addition, researchers must validate measurement tools for identifying and quantifying the severity of social impairments in the model species that has strong translational relevance for humans. The goal of the present study is not to create an animal model of autism. To the best of our knowledge, animals do not develop autism and even nonhuman primates lack many of the higher socio-cognitive skills that may be affected by ASD [3]. Rather our goal is to validate a survey-based tool for quantifying the social responsiveness of rhesus macaques based on the human Social Responsiveness Scale, or SRS [4]. Autism, however, is characterized by social impairments and evaluating whether similar impairments are present in other species can provide unique opportunities to gain insights into the biological mechanisms underlying these behaviors.

Rhesus monkeys are a widely-used and well-studied species for understanding human neuropsychiatric disorders [5]. They share many of the same social and emotional processes that are affected in these disorders, including the presence of strong maternal bonds, reciprocal social interactions, and elaborate communicative repertoires that include complex vocalizations and facial expressions [6–10]. Moreover, the rhesus monkey brain shares many of the same structural and functional neural circuits involved in processing social information as the human brain [11–14]. Similarly, the rhesus monkey autonomic nervous system (ANS) responds to social and affective information in a manner that is consistent with the human ANS [15]. Because the behavior, neurobiology, and physiology of rhesus monkeys are so similar to humans, they have strong face and construct validity for studying the biological mechanisms underlying social impairments, including some of those associated with ASD [16]. Face validity refers to the model displaying a similar phenotype to the condition being studied, while construct validity refers to those phenotypes occurring as a result of similar underlying biological mechanisms.

Over the past few decades, several studies have used nonhuman primates to understand the behavioral and socio-emotional deficits associated with ASD. These studies have focused primarily on manipulating otherwise healthy animals by damaging specific brain regions, altering early rearing experience, or using biological interventions. For example, early studies in rhesus monkeys suggested that bilateral lesions of the amygdala produced autistic-like deficits in socio-emotional processing, including reduced initiation of social contact and greater social withdrawal [17–18]. However, these studies were confounded by the subjects’ rearing history, which involved maternal separation and only limited social contact with peers. In the absence of brain lesions, it is well-known that rearing manipulations produce profound social impairments in nonhuman primates. Peer-reared monkeys display blunted or atypical reactions to emotionally provocative stimuli [19], in addition to specific deficits in social cognition, including inappropriate or incompetent responses to social situations, self-injurious behavior, increased motor stereotypies, heightened stress responses, and substance abuse [20]. These behavioral impairments, in addition to concomitant deficits in overall HPA axis function, are common to many neuropsychiatric conditions and do not appear to have strong face validity for ASD. Moreover, when amygdala lesions were performed on neonates that were subsequently reared by their mothers in more enriched social environments, the early variation in social and communicative behavior [21] normalized with age [22–23]. Broad social deficits in infant monkeys have also been reported in studies that manipulated the immune environment of pregnant females. Infants showed deficits in social attention, increased repetitive behaviors, reduced affiliative vocalizations, inappropriate social interactions with unfamiliar conspecifics, and altered dendritic morphology in prefrontal cortex [24–26]. However, these impairments only occurred when the subject was removed from their familiar home environment and tested either alone or with unfamiliar conspecifics [24]. While it is clear that these studies have been successful at producing social impairments of various types and degrees, their face and construct validity is weak with regard to the core social impairments associated with ASD.

We propose that a great deal can be learned about the biological mechanisms underlying social impairments by studying naturally-occurring variability in social behavior. This is an important translational approach because the core social impairments exhibited by individuals with ASD are not absent in the general population, but instead are behaviors that are represented at the extreme ends of a distribution of traits [27–29]. In humans, these traits can be measured using an easy to implement, 65-question survey called the Social Responsiveness Scale, or SRS [30]. The survey questions are organized into subcategories that separately address the domains of receptive, cognitive, expressive, and motivational aspects of social behavior. Importantly, the factor structure derived from the SRS reveals a single dimension that contains items/questions that map to each of ASD’s core symptoms. Therefore, the tool is able to capture variability in traits that are associated across different behavioral and social domains [31]. Moreover, the scores derived from the SRS show a unimodal distribution within the human population, including both children and adults which enables researchers to quantify various levels of social responsiveness, ranging broadly from typical responsiveness to a diagnosis of ASD [30].

The SRS differs considerably from existing tools for diagnosing ASD, such as the Autism Diagnostic Interview-Revised, or ADI-R [32], which is a semi-structured parent interview, or the Autism Diagnostic Observational Schedule, or ADOS [33], which involves direct observation of the child during semi-structured social interactions. The ADI-R and ADOS provide categorical assessments of social impairment in order to confirm a diagnosis of ASD, and this diagnosis is highly correlated with SRS scores [30, 34–36]. However, because the SRS produces a unimodal distribution of scores, it can be used to identify individuals who display atypical behaviors in comparison to the general population, but who do not reach the diagnostic threshold for ASD [37]. For example, genetic susceptibility studies have demonstrated the heritability of autistic traits by using the SRS to identify similar behaviors in the relatives of those diagnosed with ASD, albeit at a weaker, or subthreshold, level of severity [37]. This reinforces the idea that autistic behaviors are present within the general population, albeit at unusually high or low frequencies. Thus, the SRS has the advantage of being able to identify both typical *and* atypical variation in these traits [4, 27–29]. In sum, these findings demonstrate the ability of the SRS to identify a distribution of behavioral traits in the general population that covary with ASD symptomatology.

The SRS would be an extremely useful tool for studies aimed at understanding the behavior and neurobiology of social impairments, and for evaluating their potential treatment. Not only does it provide a quantitative measure of behavioral variability, but it has strong translational relevance for human neuropsychiatric conditions, particularly ASD. Previous studies have successfully adapted the SRS for use in the chimpanzee, indicating that it may be adaptable to laboratory species commonly used in biobehavioral research, such as the rhesus monkey [38]. Starting with the original 65-item SRS [4], Marrus and colleagues first removed questions that specifically related to language (e.g. “takes things too literally and doesn’t get the real meaning of a conversation”), or would have been difficult to interpret across species (e.g. “can’t get something off his or her mind when he or she starts thinking about it”). Next, they altered some item wordings to reflect species-specific chimpanzee behavior patterns (e.g. such as grooming style). The resulting chimpanzee SRS contained 36 items and was given to chimpanzee caretakers to complete on 29 chimpanzees living at three different sites. Like the human SRS, responses on this tool revealed a continuous distribution of scores and a unitary factor structure that accounted for over 52% of the variance. Four items were strongly correlated with the primary factor and, as with the human SRS, these items represented behaviors associated with each of the core symptoms of ASD, social, communicative and repetitive behaviors. The authors then cross-validated this tool by giving it back to human children both with and without ASD and confirmed that it was still accurately able to categorize individuals based on diagnosis. This suggests strong face validity in that the tool appears to measure a similar phenotype across two related species of primates. However, despite these findings, cost and ethical concerns preclude chimpanzees from serving as a viable animal species for studying human disease. In contrast, the rhesus monkey is an excellent species in which to model the biological mechanisms underlying human neuropsychiatric conditions, including many of the social impairments associated with ASD. Therefore, it was the goal of this study to adapt the chimpanzee SRS for use in the rhesus monkey and perform a series of initial assessments to validate the basic structure of the tool for monkeys. Such a validation would represent a significant methodological advancement in the development of translational animal models for understanding the biological mechanisms underlying social impairments.

## Methods

### The mSRS Development

The primary goal of this study was to make the initial steps towards developing a survey-based tool for assessing variability in the social responsiveness of rhesus macaques that could translate to humans. The first step was to determine how well the 36-item chimpanzee SRS [38] would translate to the rhesus monkey. For this initial study, the only changes to the existing chimpanzee SRS were to change the word ‘chimpanzee’ to ‘macaque’ and modify a few other questions to emphasize macaque-specific behavior patterns. For example, item #25 of the chimpanzee SRS asks whether the individual ‘has repetitive, odd behaviors such as hand flapping, rocking/swaying’ etc., to which we added ‘tumbling or spinning’ based on the propensity of macaques to engage in this type of acrobatic behavior, especially as infants and juveniles. For some questions, we added the clarification ‘…for individuals of that age, rank, or gender.’ This was important because one of the advantages of using an SRS tool for assessing social impairment is to try and capture that impairment at early time points to identify individuals that could be targeted for intervention. Marrus and colleagues [38], however, only validated the chimpanzee SRS in adults. Moreover, recent studies have found rank differences in the SRS scores in chimpanzees [39]. The social organization of the rhesus monkey is based around a linear dominance hierarchy and is, therefore, considerably different from that of chimpanzees and humans. The tool that reflects these changes is referred to as the mSRS for macaque SRS (see Supplementary Material S1 Text).

The mSRS was converted into a Google document so that it could be completed by raters and submitted online. For each survey form, the rater was asked to provide the monkey’s identification number, age and rank, and the type of compound in which it lived. Each of the 36-questions allowed for four possible responses; 1 = not true, 2 = sometimes true, 3 = often true, or 4 = almost always true. This was based directly on the original answer format of the SRS [30], and its chimpanzee adaptation [38]. There was no option for “I don’t know,” but raters were told to skip any question that they felt they could not adequately answer. In order to avoid response biases, questions were worded in both frequent and infrequent directions: for example, “the monkey often exhibits this behavior” and “the monkey rarely exhibits this behavior.” Question responses were reverse-scored prior to the final summary such that higher scores were related to greater social impairment [30]. The final summed mSRS scores could range between 0 and 108.

Although this study did not involve any direct manipulation or use of animals, the animals that were the focus of the survey ratings were housed and cared for in strict accordance with the recommendations in the Guide for the Care and Use of Laboratory Animals of the National Institutes of Health. (**S1 Text. The macaque social responsiveness scale**)

### Raters and Subjects

Potential raters included individuals currently employed in either the research or colony management units at the Yerkes National Primate Research Center (YNPRC) in Lawrenceville, GA. These individuals were emailed a confidential request to complete surveys on the behavioral traits of monkeys living at the YNPRC for which they had worked with for at least 6 months. This time frame was arbitrarily set to standardize the experience of the raters for this initial study. The email outlined the purpose of the study and gave basic instructions for completing the monkey surveys. Raters were also instructed to not discuss their responses with other raters. Consent to participate was voluntary and was documented by a confidential reply to the initial email. Potential raters could either choose not to respond to the email, or respond negatively, as a means to opt-out. The raters who agreed to participate in this study were then emailed the Google document containing the 36-item mSRS. The surveys included a space for the rater to provide their name so that raters could be dissociated for the purpose of data analysis. Information about the raters’ years of experience working with monkeys was also requested. Raters were not required to submit their full name, nor any personal health information. After the survey collection period but prior to all analyses, both the macaque and human identifiers were anonymized by the lead author by converting them to numerical form.

Additionally, an analysis of inter-rater and intra-item reliability requires the same monkeys to be rated by the same set of raters. To achieve this, additional ratings were obtained from four raters who all worked with a separate group of 16 monkeys that were part of an unrelated study approved by the IACUC of Emory University (protocol number 2002763). The raters’ experience with these monkeys consisted of 2–3 months of daily accesses, hands-on training, and casual observation.

The YNPRC field station houses approximately 3000 socially housed monkeys that live mostly in large indoor-outdoor compounds. The YNPRC has been continuously and fully accredited by the Association for the Assessment and Accreditation of Laboratory Animal Care International (AAALAC) for the last 32 years. Animals receive complete professional medical care from members of fully staffed veterinary medicine and colony management departments. No restrictions were given as to which monkeys could be rated, so surveys were completed for males and females, both living and deceased, covering a broad range of ages, ranks, rearing experience, housing conditions, and experimental histories. It was not the goal of the present study to examine the effects of all conditions on the mSRS scores, only the effects of age, gender and rank to provide data comparable to the human and chimpanzee SRS. Unless they were actively assigned to a research protocol approved by the Institutional Animal Care and Use Committee (IACUC) of Emory University during the period in which the raters were familiar with them, all of the animals rated for this study received ad libitum water, chow twice daily, and daily enrichment that included both edible and destructible forage. This study did not involve any active observation or experimentation on any living monkey and thus IACUC approval was not required.

### Data Analysis

In order to validate the mSRS for assessing variability in the social responsiveness of rhesus macaques and to compare it to the SRS and the chimpanzee SRS, several stages of analyses were conducted. First, the overall mSRS scores were plotted and the shape of the distribution assessed for normality using Lilliefors’ test. Correlations between the mSRS scores and the rank, age and gender of the subjects were then calculated, and differences between these variables and overall mSRS scores were evaluated using a General Linear Model. Because ordinal rank has little meaning when different compounds contain a different number and composition of matrilines, rank was calculated categorically as either lower third (low), middle third (middle), or highest third (high) of the existing matrilines. Second, inter-rater reliability was evaluated using intra-class correlations (ICC), which compare the variability between different ratings of the same subject compared to the overall variability across all raters and subjects. The item-level reliability was analyzed using Chronbach’s alpha (http://www.mathworks.com/matlabcentral/fileexchange/22099-intraclass-correlation-coefficient—icc-). This requires that the same monkey be rated by multiple raters and thus provides an assessment of whether raters scored each question in a similar manner for the same monkey. Any individual item with a negative alpha value was excluded from the factor analysis. Questions with negative alpha values may reflect an inability of the chimpanzee SRS to translate directly to the rhesus macaque and future studies will attempt to modify these questions to be more macaque appropriate. All of the above mentioned statistics were performed using the statistical toolbox in MATLAB.

For the final analysis, an Exploratory Factor Analysis (EFA) was performed to determine the overall factor structure of the mSRS tool using the factanal() function in R. EFA is a method used to describe variability among observed variables and how these can be represented by fewer unobserved variables, referred to as factors. We used the ‘eigenvalues greater-than-one’ rule and bootstrapping to generate 95% confidence intervals (CI) for the eigenvalues to determine the number of factors to extract. Factors for which the eigenvalues had lower CI limits above 1 were included. After determining the number of factors, EFA was performed with varimax rotation. The factor loadings were bootstrapped to generate 95% confidence intervals for the loadings themselves. As a result, this procedure enabled the use of median boostrapped factor loading estimates instead of the point estimates from a single factor analysis. All bootstrap analyses included 10,000 randomizations, with replacement.

## Results

### Demographics

Fifteen raters with between 2 and 11 years of experience working with monkeys completed 175 surveys on 105 adult monkeys (91 females and 14 males) over 4 years of age. Each rater completed an average of just over 11 surveys each, but the actual number ranged broadly between 1 and 44 surveys. These monkeys lived in a variety of social groups from smaller run compounds to large social groups containing over 100 individuals. All but 2 of the individuals rated were born at the YNPRC, and rearing histories included both peer and mother rearing. For this initial paper, the effects of compound type and rearing history will not be considered. **Table 1**provides the demographic information for the sample of 105 monkeys rated at the YNPRC including their age, rank and gender.

**Table 1:** Age, rank and gender of the 105 adult monkeys.

				Rank / Age				
		High		Middle		Low		Unknown
Gender	>10 yrs	4–10 yrs	>10 yrs	4–10 yrs	>10 yrs	4–10 yrs	>10 yrs	4–10 yrs
Females	25	7	16	4	20	11	5	3
Males	3	4	1	0	3	2	0	1

### mSRS Score Distribution

**Fig 1:**
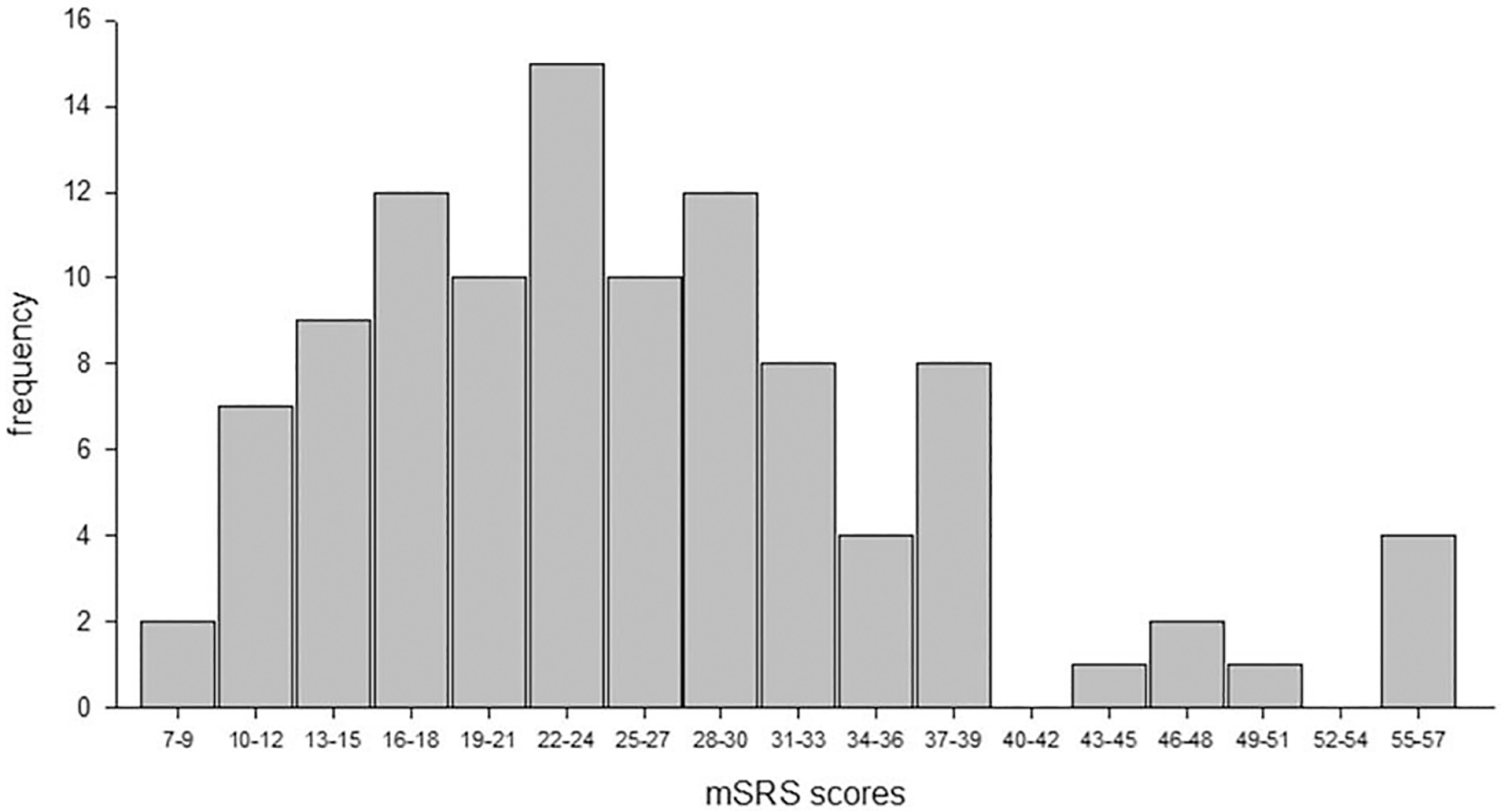
The distribution of mSRS scores for the 105 monkeys.

The overall distribution of overall mSRS scores can be seen in **Fig 1**. The mean mSRS was 25.66 with a standard deviation of 10.85. The distribution was positively skewed (skew = 0.94) and a Lilliefors test for normality confirmed that the score distribution was not normally distributed, KS = 0.10, p< 0.02. The distribution showed the presence of 6 outliers that had mSRS scores that were greater than two standard deviations above the mean. Without these outliers, the scores were normally distributed, KS = 0.05, p> 0.5.

**Fig 2:**
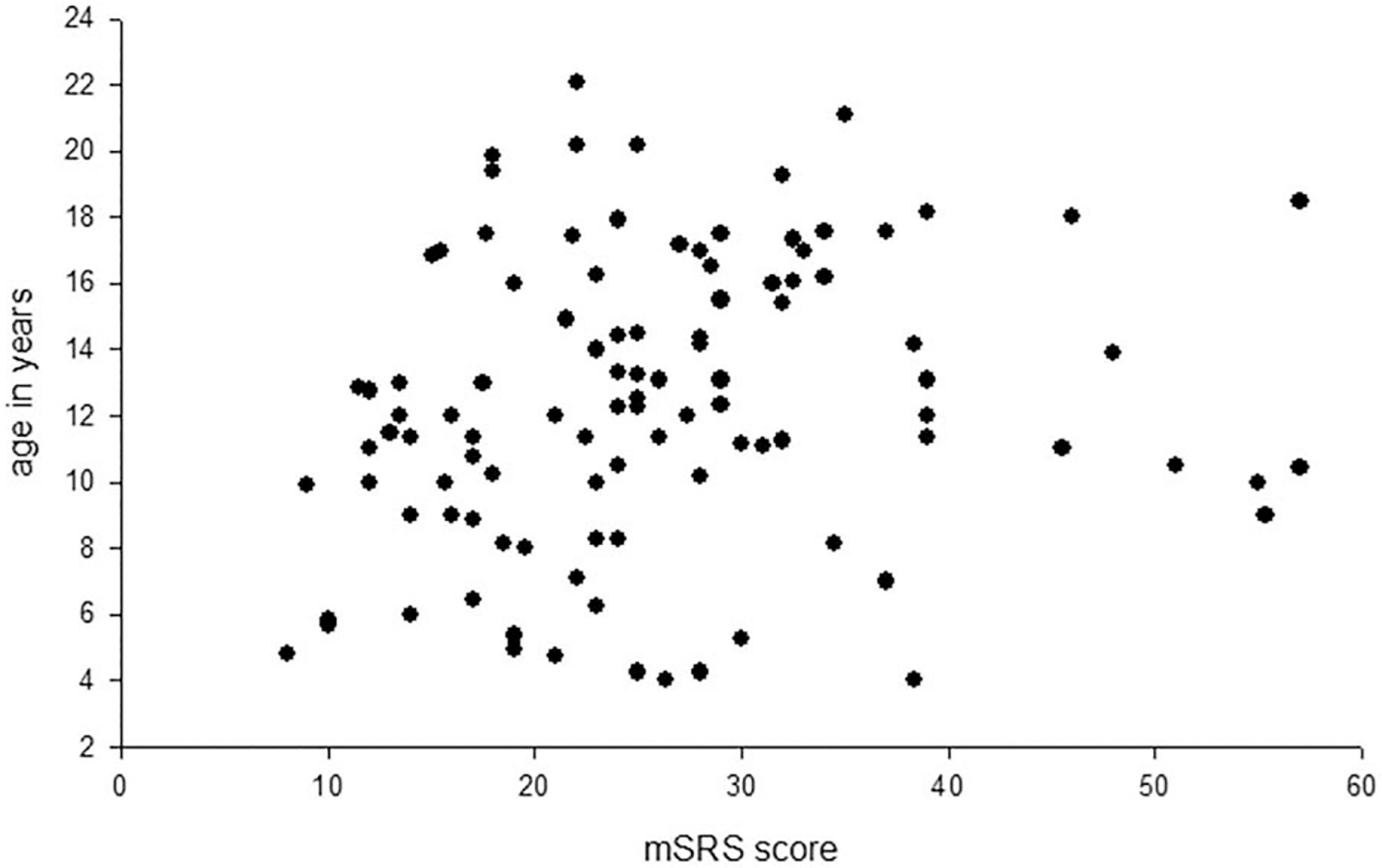
Distribution of mSRS scores by age.

**Fig 3:**
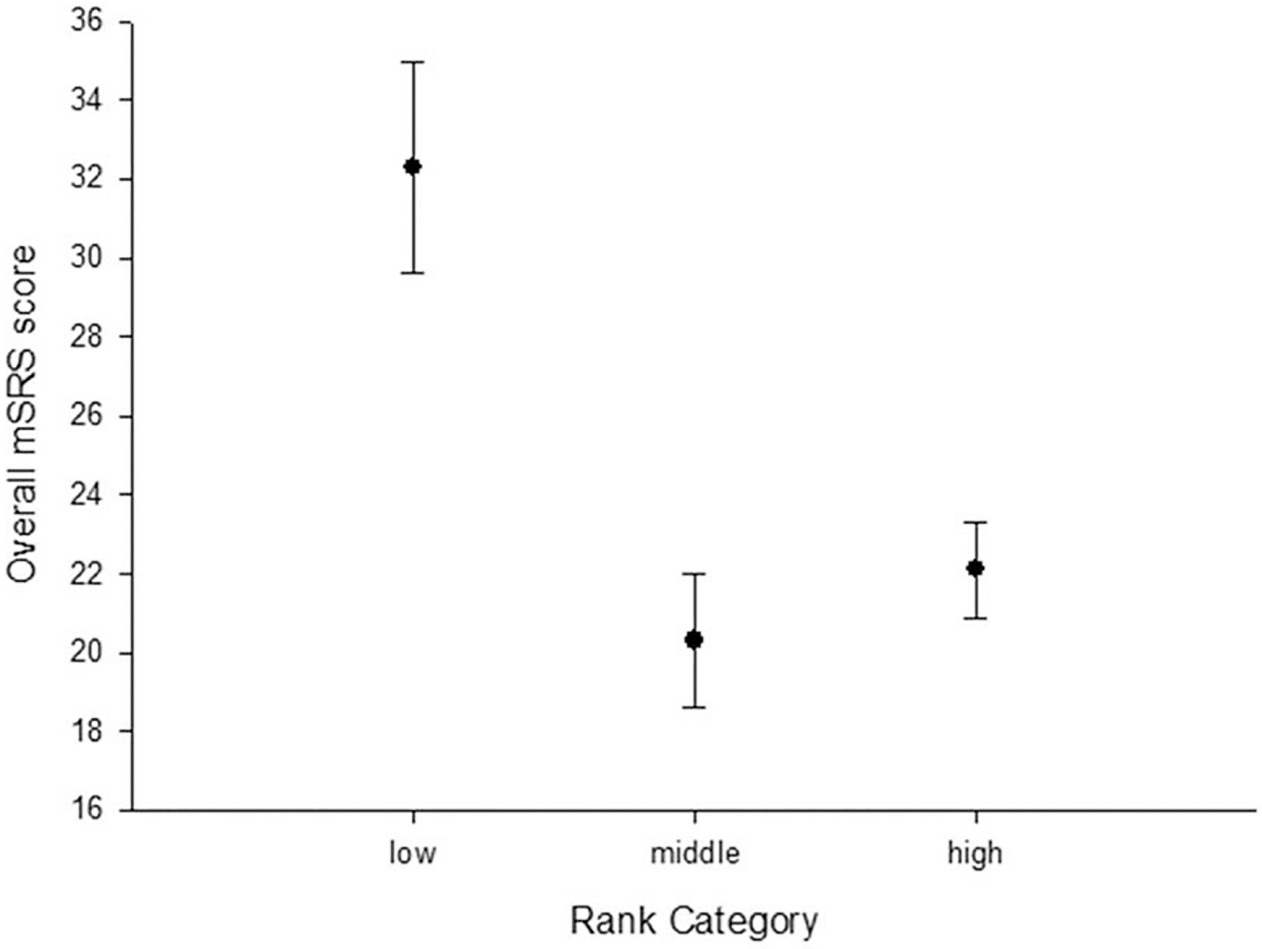
Mean (+SEM) mSRS scores for the high, middle and low ranking monkeys.

Spearman’s rank correlation analysis revealed a small, but significant, correlation between age and mSRS scores, r = 0.23, p< 0.03, where scores were higher with age (**see Fig 2**). A General Linear Model was also performed using the MATLAB statistical toolbox in which age, rank and gender were the fixed factors and the mSRS scores were the dependent variables. This confirmed the results of the t-tests described above. Overall, there was a significant main effect of age, F(66,10) = 3.14, p< 0.03, and rank, F(2,10) = 19.29, p< 0.001, but no significant main effect of gender on the mSRS scores, F(1,10) = 0.02, p = 0.89. There was also a significant gender by age interaction, F(1,10) = 6.31, p = 0.031, which can be explained by the females (mean 12.8 years) being slightly, but significantly, older than the males (mean 8.4 years) surveyed. No other interactions reached significance. **Fig 3**shows the mean mSRS values for individuals of each rank category.

### Inter-rater and Intra-item Reliability

Two-way, mixed average intra-class correlations (ICC) were used to assess the reliability of raters on the 36-item survey. Because only a few of the 15 raters provided surveys on the same monkeys, the ICC correlations could not be computed across all raters. Instead, we calculated ICC coefficients for each pair of raters that did rate the same monkeys. This amounted to 33 out of a possible 91 pairs of raters. The average ICC coefficient across all 33 pairs of raters was 0.62, which indicates substantial reliability [40]. In fact, two thirds of the pairs of raters had ICCs greater than 0.6, while only 6 pairs of raters had ICCs less than 0.4. Only one pair of raters had a negative ICC value, indicating that they were unreliable with each other, but these raters were not removed from the analysis because they were each reliable with at least one other rater.

A follow-up analysis of inter-rater and intra-item reliability was also performed using four raters who each rated the same group of 16 adult female monkeys that were not part of the original sample of 105 monkeys. The raters’ experience with these monkeys consisted of 2–3 months of daily accesses, hands-on training, and casual observation. The average ICC coefficient across the 4 raters was 0.86 (SD = 0.05). The average ICC coefficients for the pairwise inter-rater reliability measures were; rater 1 vs rater 2 = 0.89, rater 1 vs rater 3 = 0.92, rater 1 vs rater 4 = 0.83, rater 2 vs rater 3 = 0.89, rater 2 vs rater 4 = 0.79, rater 3 vs rater 4 = 0.82. This reflects substantial reliability and is consistent with our findings across the 15 YNPRC raters described above.

**Fig 4:**
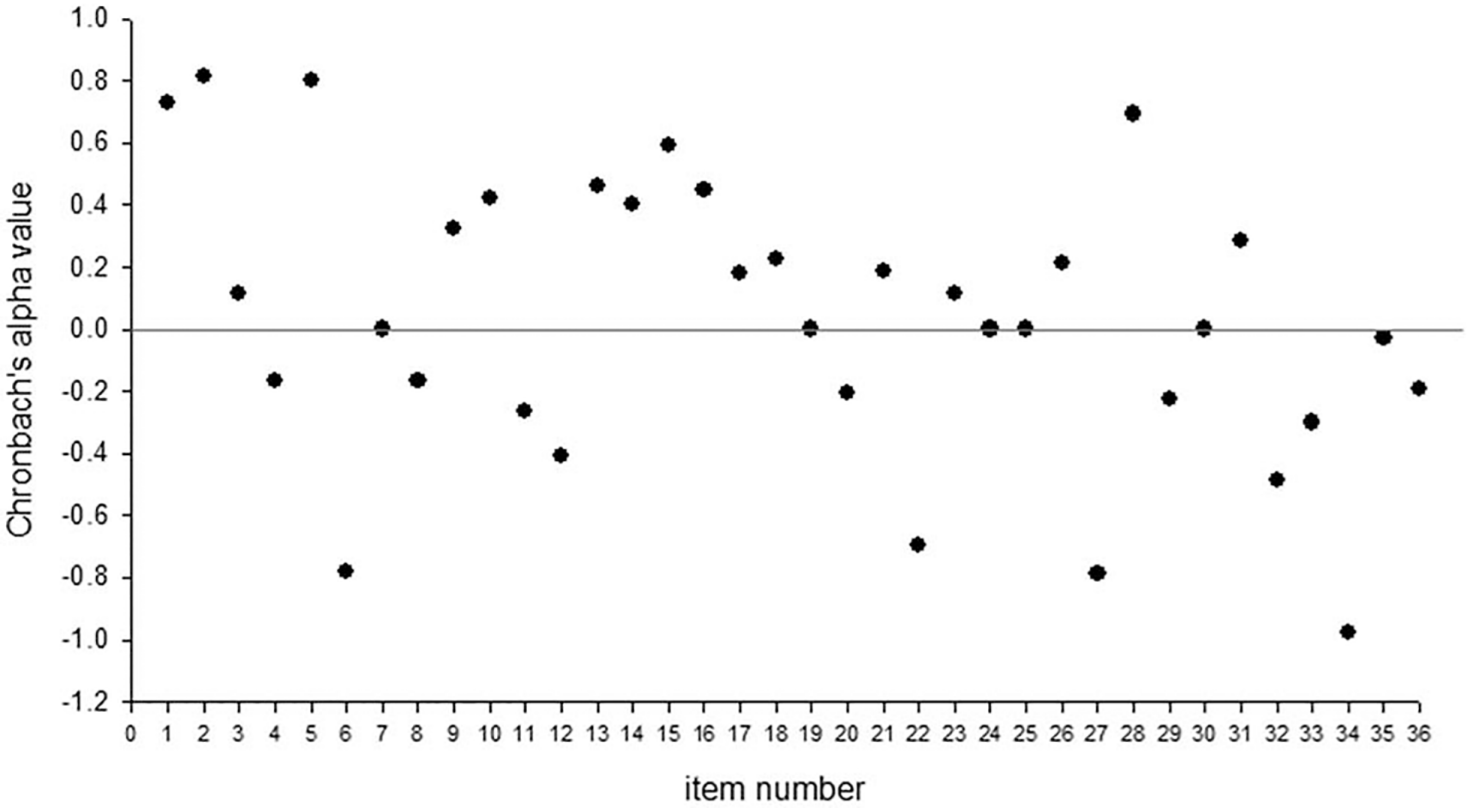
Chronbach’s alpha values for intra-item reliability in a subset of 4 raters.

An item-level analysis of the data from these 4 raters using Chronbach’s alpha showed strong reliability in some items, but also inconsistency in the reliability of other items. Seventeen items had positive alpha values, indicating some consistency in raters’ responses on these items (**see Fig 4**, and notations in S1 Text). Four of these had alpha values greater than 0.6, items 1, 2, 5 and 28, indicating substantial reliability. Five questions had alpha values between 0.4 and 0.6, items 10 and 13–16, indicating at least moderate reliability. Items 7, 19, 24, 25 and 30 showed no variance across the 16 monkeys, and fourteen items had negative values, indicating no internal consistency, including items 4, 6, 8, 11–12, 20, 22, 27, 29, 32–36. Questions that showed no variance or had negative reliability ICCs were excluded from the factor analysis described below.

### Factor Analysis

Exploratory Factor Analysis (EFA) was performed on the raw scores for the 17 items that had positive Chronbach’s alpha values from the intra-item reliability analysis described above. This was done using the factanal() function in R statistical computing language. Three factors had eigenvalues significantly greater than 1.0 and these factors accounted for 30.64% (95% CI: 20.51% - 39.06%), 17.46% (95% CI: 13.31% - 22.67%), and 11.96% (95% CI: 5.72% - 17.90%) of the variance, respectively. An evaluation of the 95% CIs derived from the bootstrapped analysis determined which items loaded significantly onto which factors. The median factor loadings and their 95% CIs for the 17 items are presented in **Table 2**. Items that loaded significantly on Factor 1 were 1–3, 5, 9–10, 13, 15, 17–18, 21, and 31 (see S1 Text for specific questions). No items loaded significantly on Factors 2 or 3, suggesting that the sample size was too small to reliably capture the latent constructs associated with these factors. Factor 1 contained items related to social avoidance (e.g. item 3, “would rather be alone than with others,” or item 15, “avoids starting social interactions with others”) in addition to social anxiety/inflexibility (e.g. item 1, “seems much more fidgety in social situations than when alone,” or item 31, “is too tense in social situations”). Negatively associated with Factor 1 were items related to social confidence (e.g. item 2, “seems self-confident when interacting with others”).

**Table 2:** Median factor loadings and the upper and lower 95% Confidence Intervals from the bootstrapped Explanatory Factor Analysis using varimax rotation. Items in bold load significantly to that factor.

					Factor Loadings				
		Factor 1			Factor 2			Factor 3	
Question #	Lower	Median	Upper	Lower	Median	Upper	Lower	Median	Upper
1	0.39	**0.63**	0.82	-0.47	0.46	0.69	-0.4	0.32	0.6
2	-0.64	**-0.37**	-0.15	-0.91	-0.63	0.88	-0.81	-0.06	0.83
3	0.24	**0.83**	0.96	-0.19	0.17	0.82	-0.22	0.09	0.68
5	-0.56	**-0.3**	-0.12	-0.96	-0.8	0.94	-0.92	-0.12	0.91
9	0.35	**0.65**	0.83	-0.38	0.36	0.63	-0.34	0.23	0.55
10	0.37	**0.74**	0.89	-0.33	0.31	0.71	-0.31	0.13	0.59
13	0.12	**0.48**	0.81	-0.16	0.14	0.73	-0.21	0.18	0.7
14	-0.44	-0.26	0	-0.4	-0.07	0.18	-0.49	-0.07	0.27
15	0.3	**0.71**	0.85	-0.31	0.27	0.68	-0.26	0.12	0.52
16	-0.11	0.18	0.68	-0.2	0.14	0.72	-0.27	0.47	0.84
17	0.47	**0.75**	0.91	-0.28	0.31	0.71	-0.25	0.29	0.67
18	0.24	**0.81**	0.95	-0.14	0.12	0.81	-0.17	0.1	0.61
21	0.19	**0.51**	0.81	-0.22	0.22	0.76	-0.2	0.33	0.8
23	-0.05	0.21	0.49	-0.26	0.01	0.44	-0.23	0.15	0.65
26	-0.25	-0.03	0.18	-0.25	0.01	0.35	-0.25	0.23	0.97
28	-0.24	-0.02	0.16	-0.75	-0.47	0.75	-0.65	0.02	0.74
31	0.12	**0.37**	0.7	-0.6	0.53	0.71	-0.53	0.29	0.63

## Discussion

The results of this initial study provide encouraging support for the development of a social responsiveness survey for use in the rhesus macaque (mSRS). Several key features of the mSRS score distribution were consistent with that found in both humans and chimpanzees on their comparable species-specific tools. First, the distribution of overall mSRS scores for the 105 monkeys was unimodal, although not normally distributed. In fact, similar to humans [41], the distribution of scores had a moderate to substantial positive skew which was driven by the presence of six outliers that had mSRS scores that were more than 2 standard deviations above the distribution mean. Without these six outliers, the scores were normally distributed. These six outliers were scored consistently by the four raters on 8 of the 12 items that were significantly associated with Factor 1 of the EFA. This factor accounted for the greatest proportion of variance in mSRS scores. Therefore, the mSRS appears capable of identifying a substantially large group of outliers, 5.7% of the overall sample size, that not only had overall mSRS scores that were considerable larger than the general population, but they received consistently high scores on the majority of individual items that loaded significantly to the primary factor. Interestingly, four of these outliers were peer-reared monkeys, a condition well-known for affecting the social competence of captive primates [20]. The influence of rearing condition on mSRS scores was not addressed in this study but will be the subject of future analyses. Second, as was the case for chimpanzees [38], no differences were observed in mSRS scores between males and females, however, males only represented 13.3% of the sample in this initial study. It should be noted that in humans, the higher SRS scores reported for males compared to females are well within what is considered a normal limit, falling close to the median value for the distribution [42]. Third, similar to a report in chimpanzees [39], there was a significant relationship between rank and mSRS scores, where higher scores were found for the lower ranking individuals. This may reflect greater social anxiety or reduced self-confidence in these individuals compared to those of higher rank, particularly given the strict, hierarchical nature of rhesus macaque societies ([43], see also [44]). These results support other lines of research that have focused on low-ranked monkeys as a model for social stress [45–46]. The current results suggest that lower ranking individuals have poorer levels of social responsiveness than higher ranking monkeys, but whether a causal relationship exists between social responsiveness and rank remains unknown. The mSRS scores in the current study had a small but significant correlation with age, with higher scores in older individuals. Although no such correlations were observed in chimpanzees, a recent study in humans demonstrated a small effect of age, such that older subjects had slightly higher SRS scores [47]. Because the mSRS and chimpanzee SRS were all derived from the human child SRS, these findings may reflect behavioral differences between children and adults and that some questions may not be phrased appropriately for older individuals. Alternatively, older individuals may be seen as more socially anxious or avoidant, perhaps as a means to avoid attention within an aggressive rhesus monkey society.

Responses on the mSRS tool showed strong and consistent reliability among the raters who provided responses for the same monkeys. Reliability was also very high among the subset of four raters who each assessed the same set of 16 additional monkeys. This indicates that the raters were assessing the social responsiveness of the monkeys, through the completion of the mSRS tool, in a similar fashion. In the current study, raters were required to have at least 6 months of experience with each individual monkey before completing the survey. However, experience can also be assessed at the species-level, i.e., how much experience a rater has with macaques in general. The current group of raters as a whole had an average of over six years of experience observing and working with monkeys when these ratings took place in the summer of 2015. This raises the question of how much experience is necessary to be a good rater on the mSRS tool. Although 6 months is not an extremely long period of time, the current set of raters were extremely experienced with rhesus monkey behavior and were able to draw from this experience when evaluating the typicality of social responsiveness in individual monkeys. Future studies will attempt to compare and quantify the amount and type of experience that produces reliable ratings in order to inform, and potentially standardize, future studies using the mSRS tool. For example, is the experience of research staff different than that of colony management or veterinary staff? Is it more important to have experience at the individual or species-level, and how much experience is required to produce consistent reliability?

Because the mSRS was ultimately derived from the human SRS, and its adaptation to the chimpanzee, it was important to also evaluate the reliability of each individual question in the survey. This could only be done for the four raters who each rated the same subgroup of 16 female macaques. The results of this analysis confirmed that only about half of the 36 items (n = 17) showed positive reliability and, of these items, only four were highly reliable (with Chronbach alpha values over 0.6). These included questions 1, 2, 5 and 28. Questions 1, 2 and 5, represented items that loaded significantly onto the primary factor and were related to social anxiety/inflexibility and social confidence. Items 10, 13–16 also showed positive reliability. Only items 10, 13 and 15 were significantly loaded onto Factor 1 in the mSRS and these reflected questions related to social avoidance and inflexibility. Only one of the items that loaded significantly onto the primary factor of the mSRS was also strongly loaded onto Factor 1 of the chimpanzee SRS [38]. This was item 17, “is socially awkward.” There were 14 items that had negative reliability, which are interpreted as unreliable questions.

Because the mSRS tool was adapted directly from the chimpanzee SRS, negative reliability for these questions could reflect their lack of pertinence for macaque monkeys, as opposed to chimpanzees and humans, or the questions may not have been phrased in a manner that was easily translatable to rhesus monkeys. For example, questions 4 and 8 were not reliable items for macaques, but both of these questions loaded strongly onto Factor 1 in the chimpanzee SRS. These questions asked whether individuals showed bizarre behaviors, or responded appropriately to others’ social cues. In general, the mSRS raters did not show confidence with any questions that addressed odd or unusual behavior. In fact, one of the chimpanzees included in the previous study had adverse early life experience that may have contributed to it displaying odd stereotypies. Ratings for this individual could be responsible for the positive loading of item 4 and negative loading of item 8 in the chimpanzee Factor 1 [38]. In comparison to these results, 74% of the 36 items in the chimpanzee SRS showed positive reliability across the three sites where chimpanzees were rated. Only 4 items had negative reliability at site 1 and 5 items had negative reliability at site 1. Therefore, it appears as though the SRS translated better between humans and chimpanzees than it did between chimpanzees and rhesus macaques. This is not surprising given that chimpanzees are more closely related to humans than they are to macaques. Future studies will evaluate each of the mSRS questions that showed negative reliability in the current study in an attempt to modify or revise them to be either more suitable to macaque-specific behavior or phrased in a manner so as to be easier for raters to answer with confidence. Moreover, because the SRS tools measure raters’ impressions of social traits and not individual behaviors, it would be very important to attempt to validate the actual behaviors that correlate with or predict variation in mSRS scores. To this end, monkeys would be observed for differences in specific behaviors and these would be correlated with mSRS scores to verify that specific behavioral differences, and which ones, are associated with the outcome of the mSRS tool.

The results of the exploratory factor analysis identified three factors that together accounted for just over 60% of the total variance in the mSRS items. However, the only significant item loadings were associated with the primary factor. This is consistent with previous data showing that the SRS and chimpanzee SRS can be represented by a unitary factor structure. Individually, the majority of these items were related to questions pertaining to social avoidance (items 3, 9, 10, 15 and 18), social confidence (items 2 and 5), social anxiety/inflexibility (items 1, 13, 16, 21 and 31), and social awkwardness (item 17). The interpretation that these findings map to the core features of ASD is consistent with the results of both the SRS and chimpanzee SRS. Interestingly, the items associated with Factor 1 of the mSRS map onto three of the five SRS domains identified in a recent, large-scale study of humans, including social isolation and withdrawal, interpersonal relatedness, and insistence on sameness [47]. Although that study identified up to five factors in humans, the results of the SRS tools from all three species studied thus far, macaques, chimpanzees and humans, have identified a primary factor that accounts for the majority of the variance. In the SRS, items that load onto this primary factor are associated with each of the three core symptoms of ASD. In chimpanzees, the items that load onto the primary factor include questions pertaining to social isolation and withdrawal, in addition to strange or bizarre behavior. In the present mSRS study, items that were associated with the primary factor included questions pertaining to social avoidance, social anxiety/inflexibility, social confidence and being socially awkward.

In conclusion, the long-term goal of the mSRS project is to generate a robust, translational tool for quantifying the social responsiveness of rhesus monkeys that has strong face, construct, and potentially predictive, validity for the social impairments associated with ASD in humans. The data presented here support the initial usefulness of mSRS tool for achieving this goal. The tool was highly reliable across raters and was able to identify a large percentage of individual monkeys that displayed atypical patterns of social responsiveness, almost 6% compared to 1.5% which is the current rate of ASD diagnosed in the United States. Similar to the SRS and the chimpanzee SRS, variability in the mSRS scores was identified by a unitary factor structure containing 12 items that were related to social avoidance, social anxiety/inflexibility, lack of social confidence, and social awkwardness. These items map closely to the social impairments characterized by ASD, although they are certainly not exhaustive with regards to ASD’s symptomatology. However, the relatively poor intra-item reliability of the mSRS reported in the current study, e.g., only 47% of the items showed positive reliability, highlights species differences in the translatability of the tool from humans to chimpanzees to macaques. Further effort is required to refine the unreliable questions to be more sensitive to macaque-specific behavioral patterns, or to be phrased in such a way as to be more easily answered by those familiar with macaque behavior. Importantly, this revised mSRS tool would need to be cross-validated by giving it back to humans with and without ASD to verify that it has not lost face validity for human social behavior, similar to the approach used by Marrus and colleagues [38]. Lastly, although the long-term goal of the mSRS project is to develop translational, cross-validated tools for assessing social responsiveness in humans and macaques, the ability of these tools to identify species-differences is still very meaningful. A comparative approach provides a means to assess what aspects of the ASD symptomatology is biologically conserved across species versus what aspects may be driven by the evolution of unique biological systems in humans [3]. Therefore, the results of this study, in addition to future studies focused on refining the mSRS, could be used to identify the extent to which evolutionarily conserved biological systems underlie variability in social responsiveness. Such an approach may ultimately help to refine hypotheses regarding the etiology of the mechanisms underlying social impairments, and ultimately a means to narrow the potential range of therapeutics for treating such impairments.

## Supporting Information

S1 Data Raw mSRS scores and demographic information for subjects. (XLSX)

S1 Text The Macaque Social Responsiveness Scale (mSRS). This file contains a list of the 36 questions included in the mSRS.
(DOCX)

## References

[1] Bowers K, Lin PI, Erickson C. Pharmacogenetic medicine in autism: Challenges and opportunities. Pediatric Drugs. 2015;17:115–24. 10.1007/s40272-014-0106-0 25420674

[2] Yuen RK, Thiruvahindrapuram B, Merico D, Walker S, Tammimies K, Hoang N, et al Whole-genome sequencing of quartet families with autism spectrum disorder. Nature medicine. 2015;21(2):185–91. 10.1038/nm.3792 25621899

[3] Penn DC, Holyoak KJ, Povinelli DJ. Darwin's mistake: explaining the discontinuity between human and nonhuman minds. The Behavioral and brain sciences. 2008;31(2):109–30; discussion 30–78. 10.1017/S0140525X08003543 18479531

[4] Constantino JN, Gruber CP. Social responsiveness scale: Western Psychological Services; 2005.

[5] Phillips KA, Bales KL, Capitanio JP, Conley A, Czoty PW, t Hart BA, et al Why primate models matter. American journal of primatology. 2014;76(9):801–27. 10.1002/ajp.22281 24723482 PMC4145602

[6] Brown GR, Dixson AF. The development of behavioural sex differences in infant rhesus macaques (Macaca mulatta). Primates. 2000;41:63–77. 30545192 10.1007/BF02557462

[7] Beisner BA, McCowan B. Signaling context modulates social function of silent bared-teeth displays in rhesus macaques (Macaca mulatta). American journal of primatology. 2014;76(2):111–21. 10.1002/ajp.22214 24038330 PMC3919452

[8] Hinde RA, Rowell TE. Communication by postures and facial expressions in the rhesus monkey (Macaca mulatta). Proceedings of the Zoological Society of London. 1962;138:1–21.

[9] Parr LA. The evolution of face processing in primates. Philos Trans R Soc Lond B Biol Sci. 2011;366(1571):1764–77. 10.1098/rstb.2010.0358 21536559 PMC3130377

[10] Thierry B. Social development in three species of macaque (Macaca mulatta, M. fascicularis, M. tonkeana): A preliminary report on the first ten weeks of life. Behav Processes. 1985;11(1):89–95. 10.1016/0376-6357(85)90105-6 24924364

[11] Kamphuis S, Dicke PW, Thier P. Neuronal substrates of gaze following in monkeys. The European journal of neuroscience. 2009;29(8):1732–8. 10.1111/j.1460-9568.2009.06730.x 19385988

[12] Miranda-Dominguez O, Mills BD, Grayson D, Woodall A, Grant KA, Kroenke CD, et al Bridging the gap between the human and macaque connectome: a quantitative comparison of global interspecies structure-function relationships and network topology. The Journal of neuroscience: the official journal of the Society for Neuroscience. 2014;34(16):5552–63. 10.1523/JNEUROSCI.4229-13.2014 PMC3988411 24741045

[13] Vincent JL, Patel GH, Fox MD, Snyder AZ, Baker JT, Van Essen DC, et al Intrinsic functional architecture in the anaesthetized monkey brain. Nature. 2007;447(7140):83–6. 17476267 10.1038/nature05758

[14] Wedeen VJ, Rosene DL, Wang R, Dai G, Mortazavi F, Hagmann P, et al The geometric structure of the brain fiber pathways. Science. 2012;335(6076):1628–34. 10.1126/science.1215280 22461612 PMC3773464

[15] Bliss-Moreau E, Machado CJ, Amaral DG. Macaque cardiac physiology is sensitive to the valence of passively viewed sensory stimuli. PloS one. 2013;8(8):e71170 10.1371/journal.pone.0071170 23940712 PMC3734104

[16] Ruhela RK, Prakash A, Medhi B. An urgent need for experimental animal model of autism in drug development. Annals of neurosciences. 2015;22(1):44–9. 10.5214/ans.0972.7531.220210 26124551 PMC4410529

[17] Bachevalier J. Medial temporal lobe structures and autism: a review of clinical and experimental findings. Neuropsychologia. 1994;32(6):627–48. 8084420 10.1016/0028-3932(94)90025-6

[18] Bachevalier J, Malkova L, Mishkin M. Effects of selective neonatal temporal lobe lesions on socioemotional behavior in infant rhesus monkeys (Macaca mulatta). Behavioral neuroscience. 2001;115(3):545–59. 11439445 10.1037//0735-7044.115.3.545

[19] Parr LA, Winslow JT, Davis M. Rearing experience differentially affects somatic and cardiac startle responses in rhesus monkeys (Macaca mulatta). Behavioral neuroscience. 2002;116(3):378–86. 12049318 10.1037//0735-7044.116.3.378

[20] Nelson EE, Winslow JT. Non-human primates: model animals for developmental psychopathology. Neuropsychopharmacology: official publication of the American College of Neuropsychopharmacology. 2009;34(1):90–105. 18800061 10.1038/npp.2008.150

[21] Bauman MD, Lavenex P, Mason WA, Capitanio JP, Amaral DG. The development of mother-infant interactions after neonatal amygdala lesions in rhesus monkeys. The Journal of neuroscience: the official journal of the Society for Neuroscience. 2004;24(3):711–21. 10.1523/JNEUROSCI.3263-03.2004 PMC6729254 14736857

[22] Bliss-Moreau E, Moadab G, Bauman MD, Amaral DG. The impact of early amygdala damage on juvenile rhesus macaque social behavior. Journal of cognitive neuroscience. 2013;25(12):2124–40. 10.1162/jocn_a_00483 24047387 PMC4330965

[23] Moadab G, Bliss-Moreau E, Amaral DG. Adult social behavior with familiar partners following neonatal amygdala or hippocampus damage. Behavioral neuroscience. 2015;129(3):339–50. 10.1037/bne0000062 26030432 PMC4452996

[24] Bauman MD, Iosif AM, Smith SE, Bregere C, Amaral DG, Patterson PH. Activation of the maternal immune system during pregnancy alters behavioral development of rhesus monkey offspring. Biological psychiatry. 2014;75(4):332–41. 10.1016/j.biopsych.2013.06.025 24011823 PMC6782053

[25] Machado CJ, Whitaker AM, Smith SE, Patterson PH, Bauman MD. Maternal immune activation in nonhuman primates alters social attention in juvenile offspring. Biological psychiatry. 2015;77(9):823–32. 10.1016/j.biopsych.2014.07.035 25442006 PMC7010413

[26] Weir RK, Forghany R, Smith SE, Patterson PH, McAllister AK, Schumann CM, et al Preliminary evidence of neuropathology in nonhuman primates prenatally exposed to maternal immune activation. Brain, behavior, and immunity. 2015;48:139–46. 10.1016/j.bbi.2015.03.009 25816799 PMC5671487

[27] Constantino JN, Przybeck T, Friesen D, Todd RD. Reciprocal social behavior in children with and without pervasive developmental disorders. J Dev Behav Pediatr. 2000;21(1):2–11. 10706343 10.1097/00004703-200002000-00002

[28] Constantino JN, Todd RD. Autistic traits in the general population: a twin study. Arch Gen Psychiatry. 2003;60(5):524–30. 12742874 10.1001/archpsyc.60.5.524

[29] Piven J, Palmer P, Jacobi D, Childress D, Arndt S. Broader autism phenotype: evidence from a family history study of multiple-incidence autism families. The American journal of psychiatry. 1997;154(2):185–90. 9016266 10.1176/ajp.154.2.185

[30] Constantino JN, Davis SA, Todd RD, Schindler MK, Gross MM, Brophy SL, et al Validation of a brief quantitative measure of autistic traits: comparison of the social responsiveness scale with the autism diagnostic interview-revised. Journal of autism and developmental disorders. 2003;33(4):427–33. 12959421 10.1023/a:1025014929212

[31] Constantino JN, Gruber CP, Davis S, Hayes S, Passanante N, Przybeck T. The factor structure of autistic traits. Journal of child psychology and psychiatry, and allied disciplines. 2004;45(4):719–26. 15056304 10.1111/j.1469-7610.2004.00266.x

[32] Lord C, Rutter M, Le Couteur A. Autism Diagnostic Interview-Revised: a revised version of a diagnostic interview for caregivers of individuals with possible pervasive developmental disorders. Journal of autism and developmental disorders. 1994;24(5):659–85. 7814313 10.1007/BF02172145

[33] Lord C, Risi S, Lambrecht L, Cook EH Jr., Leventhal BL, DiLavore PC, et al The autism diagnostic observation schedule-generic: a standard measure of social and communication deficits associated with the spectrum of autism. Journal of autism and developmental disorders. 2000;30(3):205–23. 11055457

[34] Bolte S, Poustka F, Constantino JN. Assessing autistic traits: cross-cultural validation of the social responsiveness scale (SRS). Autism Res. 2008;1(6):354–63. 10.1002/aur.49 19360690

[35] Duvekot J, van der Ende J, Verhulst FC, Greaves-Lord K. The Screening Accuracy of the Parent and Teacher-Reported Social Responsiveness Scale (SRS): Comparison with the 3Di and ADOS. Journal of autism and developmental disorders. 2014. 10.1007/s10803-014-2323-3 25428292

[36] Takei R, Matsuo J, Takahashi H, Uchiyama T, Kunugi H, Kamio Y. Verification of the utility of the social responsiveness scale for adults in non-clinical and clinical adult populations in Japan. BMC Psychiatry. 2014;14:302 10.1186/s12888-014-0302-z 25403232 PMC4237729

[37] Constantino JN, Lajonchere C, Lutz M, Gray T, Abbacchi A, McKenna K, et al Autistic social impairment in the siblings of children with pervasive developmental disorders. The American journal of psychiatry. 2006;163(2):294–6. 16449484 10.1176/appi.ajp.163.2.294

[38] Marrus N, Faughn C, Shuman J, Petersen SE, Constantino JN, Povinelli DJ, et al Initial description of a quantitative, cross-species (chimpanzee-human) social responsiveness measure. J Am Acad Child Adolesc Psychiatry. 2011;50(5):508–18. 10.1016/j.jaac.2011.01.009 21515200 PMC3082744

[39] Faughn C, Marrus N, Shuman J, Ross SR, Constantino JN, Pruett JR Jr, et al Brief Report: Chimpanzee Social Responsiveness Scale (CSRS) Detects Individual Variation in Social Responsiveness for Captive Chimpanzees. Journal of autism and developmental disorders. 2015;45(5):1483–8. 10.1007/s10803-014-2273-9 25312279 PMC5503195

[40] Landis JR, Koch GG. The measurement of observer agreement for categorical data. Biometrics 1977;33:159–74. 843571

[41] Kamio Y, Inada N, Moriwaki A, Kuroda M, Koyama T, Tsujii H, et al Quantitative autistic traits ascertained in a national survey of 22 529 Japanese schoolchildren. Acta Psychiatr Scand. 2013;128(1):45–53. 10.1111/acps.12034 23171198 PMC3604131

[42] Hus V, Bishop S, Gotham K, Huerta M, Lord C. Factors influencing scores on the social responsiveness scale. Journal of child psychology and psychiatry, and allied disciplines. 2013;54(2):216–24. 10.1111/j.1469-7610.2012.02589.x 22823182 PMC3504640

[43] Vessey SH. Dominance among rhesus monkeys. Political Psychology. 1984;5:623–8.

[44] Fushing H, McAssey MP, Beisner B, McCowan B. Ranking network of a captive rhesus macaque society: a sophisticated corporative kingdom. PloS one. 2011;6(3):e17817 10.1371/journal.pone.0017817 21423627 PMC3058001

[45] Michopoulos V, Reding KM, Wilson ME, Toufexis D. Social subordination impairs hypothalamic-pituitary-adrenal function in female primates. Hormones and behavior. 2012;62:389–99. 10.1016/j.yhbeh.2012.07.014 22940527 PMC3477274

[46] Sapolsky RM. The influence of social hierarchy on primate health. Science. 2005;308(5722):648–52. 15860617 10.1126/science.1106477

[47] Frazier TW, Ratliff KR, Gruber C, Zhang Y, Law PA, Constantino JN. Confirmatory factor analytic structure and measurement invariance of quantitative autistic traits measured by the social responsiveness scale-2. Autism: the international journal of research and practice. 2014;18(1):31–44. 24019124 10.1177/1362361313500382

