# Correction: The Macaque Social Responsiveness Scale (mSRS): A Rapid Screening Tool for Assessing Variability in the Social Responsiveness of Rhesus Monkeys (*Macaca mulatta*)

**DOI:** 10.1371/journal.pone.0152644

**Published:** 2016-03-24

**Authors:** Eric J. Feczko, Eliza Bliss-Moreau, Hasse Walum, John R. Pruett, Lisa A. Parr

The incorrect S1 File appears in the published paper. Please view the correct S1 Text below.

## Supporting Information

S1 Text The Macaque Social Responsiveness Scale (mSRS). This file contains a list of the 36 questions included in the mSRS. Please view the correct S1 Text here.
(DOCX)
