# Supplementary material for: The Macaque Social Responsiveness Scale (mSRS): A Rapid Screening Tool for Assessing Variability in the Social Responsiveness of Rhesus Monkeys (Macaca mulatta)
Source: PLoS One. 2016 Jan 5;11(1):e0145956. doi: 10.1371/journal.pone.0145956 (PMC4701177; doi:10.1371/journal.pone.0145956)
Supplement: S1 Text — This file contains a list of the 36 questions included in the mSRS. (DOCX) [file pone.0145956.s002.docx]

S1 Text. Social Responsiveness Survey for the Rhesus macaque

KEY

1 = not true, 2 = sometimes true, 3 = often true, 4 = almost always true

2. Seems self-confident when interacting with others.

1 _________ 2 _________ 3 _________ 4 _________

3. Would rather be alone than with others.

1 _________ 2 _________ 3 _________ 4 _________

4. Behaves in ways that seem strange or bizarre for others of comparable age/rank/gender categories (*^).

1 _________ 2 _________ 3 _________ 4 _________

7. Is not well coordinated in physical activities.

1 _________ 2 _________ 3 _________ 4 _________

8. Responds appropriately to other monkeys’ vocalizations and facial expressions (*^).

1 _________ 2 _________ 3 _________ 4 _________

9. Avoids eye contact or has unusual eye contact (*).

1 _________ 2 _________ 3 _________ 4 _________

12. Plays appropriately with peers.

1 _________ 2 _________ 3 _________ 4 _________

15. Avoids starting social interactions with others.

1 _________ 2 _________ 3 _________ 4 _________

17. Is socially awkward (*^).

1 _________ 2 _________ 3 _________ 4 _________

19. Has a restricted or unusually narrow range of interests.

1 _________ 2 _________ 3 _________ 4 _________

20. Wanders aimlessly from one activity to another.

1 _________ 2 _________ 3 _________ 4 _________

25. Has repetitive, odd behaviors such as hand flapping, rocking/swaying, tumbling or spinning (*^).

1 _________ 2 _________ 3 _________ 4 _________

30. Touches others in an unusual way, e.g., makes contact that doesn’t lead to any specific interaction like contact sit, grooming.

1 _________ 2 _________ 3 _________ 4 _________

31. Is too tense in social situations, e.g., walks stiffly, stiffens or freezes when others approach.

1 _________ 2 _________ 3 _________ 4 _________

32. Stares or gazes off into space.

1 _________ 2 _________ 3 _________ 4 _________

33. Manifests species-typical reaction to loss of a valued resource.

1 _________ 2 _________ 3 _________ 4 _________

34. Grooms a variety of individuals (shows indiscriminate grooming), not the same individual daily or throughout the day.

1 _________ 2 _________ 3 _________ 4 _________
