# Supplementary material for: Correction: The Macaque Social Responsiveness Scale (mSRS): A Rapid Screening Tool for Assessing Variability in the Social Responsiveness of Rhesus Monkeys (Macaca mulatta)
Source: PLoS One. 2016 Mar 24;11(3):e0152644. doi: 10.1371/journal.pone.0152644 (PMC4807010; doi:10.1371/journal.pone.0152644)
Supplement: S1 Text — This file contains a list of the 36 questions included in the mSRS. Please view the correct S1 Text here. (DOCX) [file pone.0152644.s001.docx]

Supplementary Material_S1. List of items included in the macaque SRS with cross-reference for the subset of items in the XSRS, items that loaded positively to Factor 1 in the chimpanzee SRS (Marrus et al., 2011), items that showed positive intra-item reliability, items that showed significant loading onto Factor 1, and items that had no variance in the current study.

____________________________________________________________________________________

Item Key

*one of the 12 items in the short XSRS

^one of the 4 items most strongly loaded to the primary factor from Marrus et al., 2011

+Positive reliability

1-Items significantly loaded on Factor 1

0-Items with no variance

____________________________________________________________________________________

1. Seems much more fidgety in social situations than when alone (+, 1).

2. Seems self-confident when interacting with others (+, 1).

3. Would rather be alone than with others (+, 1).

4. Behaves in ways that seem strange or bizarre for others of comparable age/rank/gender categories (*^).

5. Has good self-confidence (+, 1).

6. Is able to communicate his or her feelings to others with gestures (*).

7. Is not well coordinated in physical activities (0).

8. Responds appropriately to other monkeys’ vocalizations and facial expressions (*^).

9. Avoids eye contact or has unusual eye contact (*,+, 1).

10. Does not attempt to interact with other monkeys (*, +, 1).

11. Is able to imitate others’ actions.

12. Plays appropriately with peers.

13. Has more difficulty than other monkeys with changes in daily routines (*, +, 1).

14. Offers comfort to others when they are sad, e.g., with grooming or other reassuring gestures (*, +).

15. Avoids starting social interactions with others (+, 1).

16. Becomes upset in situations where there are a lot of things going on (+).

17. Is socially awkward (*^, +, 1).

18. Avoids other monkeys that may want to be emotionally close to him/her (+, 1).

19. Has a restricted or unusually narrow range of interests (0).

20. Wanders aimlessly from one activity to another.

21. Seems overly sensitive to sounds or textures (+, 1).

22. Focuses his or her attention to where others are looking or listening (*).

23. Has overly serious facial expressions, e.g., is not playful (+).

24. Is too silly or makes inappropriate noises (*, 0).

25. Has repetitive, odd behaviors such as hand flapping, rocking/swaying, tumbling or spinning (*^, 0).

26. Knows when he or she is loud and making too much noise but continues to be noisy (+).

27. Knows when he or she is too close to someone or is invading someone’s space.

28. Walks in between two monkeys or disrupts them while they are interacting (+).

29. Is emotionally distant, doesn’t show his/her feelings (*).

30. Touches others in an unusual way, e.g., makes contact that doesn’t lead to any specific interaction like contact sit, grooming (0).

31. Is too tense in social situations, e.g., walks stiffly, stiffens or freezes when others approach (+, 1).

32. Stares or gazes off into space.

33. Manifests species-typical reaction to loss of a valued resource.

34. Grooms a variety of individuals (shows indiscriminate grooming), not the same individual daily or throughout the day.

35. Other monkeys do not like to play with him/her.

36. Investigates areas or situations which are unusual, loud, and/or stimulating.
